# Repurposing Dexmedetomidine: Early Pharmacological Hypothermia Enhances Neuroprotection and Improves Locomotor and Bladder Functional Recovery after Spinal Cord Injury

**DOI:** 10.1523/JNEUROSCI.0007-26.2026

**Published:** 2026-03-20

**Authors:** Aytak Khabbaz, Lilesh Kumar Pradhan, Anne Elizabeth Gowan, Samhita Chakraborty, Yihong Zhang, Fang Yuan, Eris Deanai Harris, Wei Li, Christopher Yu, Qigui Yu, Xiang Gao, Lingxiao Deng

**Affiliations:** ^1^Department of Neurological Surgery, Indiana University School of Medicine, Indianapolis, Indiana 46202; ^2^Spinal Cord and Brain Injury Research Group, Stark Neuroscience Research Institute, Indianapolis, Indiana 46202; ^3^Department of Microbiology and Immunology, Indiana University School of Medicine, Indianapolis, Indiana 46202; ^4^College of Arts and Sciences ‘28 Hutton Honors | Biology Major, Indiana University, Bloomington, Indiana 47405

**Keywords:** anti-inflammation, bladder function, locomotor, pharmacological hypothermia

## Abstract

Spinal cord injury (SCI) causes progressive secondary damage, yet translation of hypothermia, one of the few preclinical neuroprotectants, has been limited by slow, equipment-dependent cooling that rarely meets the therapeutic window. We tested whether repurposing dexmedetomidine (Dex), an FDA-approved α_2_-agonist that blocks shivering and has intrinsic neuroprotection, could provide early pharmacological hypothermia to enhance recovery after SCI. Adult mice received moderate thoracic contusive SCI followed by intraperitoneal Dex (100 µg/kg) at 1 h postinjury. Core temperature, vital signs, and ECG were monitored for 24 h. Locomotor recovery, bladder function, tissue preservation, neuronal and axonal sparing, serotonergic circuitry, raphe activation, cytokine profiles, and ERK and RIPK1 signaling were assessed. Comparator groups included untreated injury, conventional surface cooling, hypothermia-prevention by heating, and ERK inhibition. At ambient room temperature (∼24°C), Dex induced rapid, stable moderate hypothermia (∼29–32°C for ∼16 h) without respiratory compromise or arrhythmia. This pharmacological hypothermia, combined with Dex's intrinsic actions, produced greater locomotor and bladder recovery than untreated injury or conventional cooling. Dex preserved perilesional tissue, neuronal survival, axonal integrity, and descending serotonergic input while restoring raphe activation. Mechanistically, Dex plus hypothermia synergistically suppressed acute proinflammatory cytokines, increased IL-10 at Days 7 and 14, activated early ERK-dependent survival signaling, and reduced acute RIPK1-associated injury; blocking hypothermia or ERK signaling attenuated these benefits. The neuroprotective effects of Dex were similar in both sexes. A single clinically relevant Dex dose provides dual-action therapy—pharmacological hypothermia plus intrinsic neuroprotection—offering an immediately translatable, equipment-free strategy for acute SCI and other neurotrauma.

## Significance Statement

This study identifies dexmedetomidine (Dex), an FDA-approved α_2_-agonist, as a rapid and pharmacologically driven method to induce sustained hypothermia following spinal cord injury (SCI). Compared with conventional physical cooling, which is slow, equipment-dependent, and often difficult to implement, Dex induced reliable early hypothermia and produced greater recovery of locomotor and bladder function in murine models. Dex also preserved spinal tissue and descending pathways, providing a mechanistic basis for its functional benefits. Given Dex's established clinical use and safety profile in monitored medical settings, these findings highlight Dex-induced hypothermia as a promising and highly translatable therapeutic strategy for acute SCI.

## Introduction

Spinal cord injury (SCI) causes irreversible motor, sensory, and autonomic deficits and imposes a substantial lifelong economic burden (Ahuja et al., 2017). While restoring axonal connectivity remains a major translational barrier, neuroprotection, preserving neural tissue by limiting secondary injury, is the most actionable strategy for improving acute outcomes (Ulndreaj et al., 2017). After the initial mechanical insult, a delayed cascade of secondary injury, including ischemia, ionic imbalance, excitotoxicity, hemorrhage, and inflammation, drives progressive tissue loss, cystic degeneration, and glial scarring (Oyinbo, 2011; Tran et al., 2018). Rapid interventions that blunt this secondary wave of damage offer the most realistic path to preserving function in the early phase of SCI.

Systemic hypothermia is one of the well-known neuroprotective interventions in preclinical SCI models (Vedantam and Levi, 2021), acting through diverse mechanisms that reduce metabolic stress, excitotoxicity, apoptosis, edema, and barrier disruption while supporting vascular and glial repair (Wang and Pearse, 2015). However, translation has been impeded by its narrow therapeutic window: in rodents, it must begin within ∼3 h (Kabon et al., 2003; Che et al., 2011; Wang and Pearse, 2015) postinjury, whereas clinical initiation typically occurs 6–8 h postinjury due to unavoidable trauma-care delays (Levi et al., 2009; Cappuccino et al., 2010; Dididze et al., 2013). Current cooling methods also rely on invasive or ICU-level systems and often fail to achieve rapid or reliable temperature reduction, especially in obese or resource-limited settings (Green and Howes, 2007). A pharmacological method that induces fast, controllable hypothermia in the field or early emergency care could overcome these barriers (Dietrich et al., 2011; Vedantam and Levi, 2021), yet no such strategy exists for SCI. Moreover, hypothermia alone provides only modest, transient benefits, and although hypothermia-neuroprotective drug synergistic combinations improve outcomes in other neurological injuries (Wang and Pearse, 2015), they remain largely unexplored in SCI.

Dexmedetomidine (Dex), an FDA-approved α_2_-adrenergic agonist used widely for ICU sedation, represents a compelling candidate for early neuroprotection after SCI. In neurological critical care, Dex is frequently paired with therapeutic hypothermia for conditions such as hypoxic–ischemic encephalopathy because of its hemodynamic stability, lack of respiratory depression, and favorable pharmacokinetics during cooling (Cosnahan et al., 2021; Elliott et al., 2022; Cocchi et al., 2025). Beyond sedation, Dex confers direct neuroprotection in models of stroke and myocardial ischemia, partly through anti-inflammatory mechanisms (Battaglini et al., 2022). Notably, Dex also has intrinsic thermoregulatory actions and can reduce core body temperature in certain trauma or environmental contexts without external cooling (Kim et al., 2025). However, no prior study has tested whether Dex administered acutely after SCI could induce therapeutic hypothermia and improve recovery.

We show that a single intraperitoneal dose of Dex (100 µg/kg) delivered 1 h after contusive SCI in mice induces moderate hypothermia (30–32°C) for up to 16 h at room temperature (24.5°C) without specialized equipment, offering a clinically feasible, prehospital-compatible cooling strategy. Dex-induced hypothermia significantly enhanced locomotor recovery—up to 65% over untreated mice and 2.5-fold greater than physical cooling alone—and benefits persisted for at least 6 weeks. Dex also improved autonomic outcomes, particularly bladder control, a top rehabilitation priority for people with SCI (Ditunno et al., 2008). Preventing Dex-induced hypothermia through external warming markedly reduced these gains, indicating that cooling is a primary mediator. Together with amplified neuroprotective and anti-inflammatory effects, these findings identify Dex as the first pharmacological agent capable of rapidly inducing therapeutic hypothermia after SCI while providing synergistic neuroprotection.

## Materials and Methods

### Study ethical approval

All animal procedures were IACUC-approved (Indiana University, Protocol #23170) and followed the NIH Guide for Care and Use of Laboratory Animals.

### Sex as a biological variable

Both sexes were included in the initial functional cohort (*n* = 10/sex/group). Because no sex differences in Dex response were detected, subsequent mechanistic studies used males only, consistent with the higher clinical incidence of SCI in men (Kang et al., 2017). Prespecified sex-stratified analyses and interaction terms were assessed in the functional cohort.

### Animals and housing

A total of 275 adult C57BL/6 mice (male and female, 8 weeks, 18–22 g; The Jackson Laboratory) were maintained in a temperature- and humidity-controlled vivarium (12 h light/dark) with *ad libitum* food and water. Mice were group-housed prior to surgery and singly housed thereafter as needed for postoperative monitoring.

### SCI model

Mice were anesthetized with Avertin (200 mg/kg, i.p.). The mouse vertebra was hold by the spine stabilizer consisting a U-shaped trough. The mice were placed on the Infinite Horizon impactor (Precision Systems and Instrumentation), and given a 10th vertebral level laminectomy followed by a 60 kdyn contusion; Shams received laminectomy only. Postoperatively, bladders were expressed twice daily for 14 d; then daily until spontaneous voiding returned.

### Experimental groups and study design

**Figure 1.**
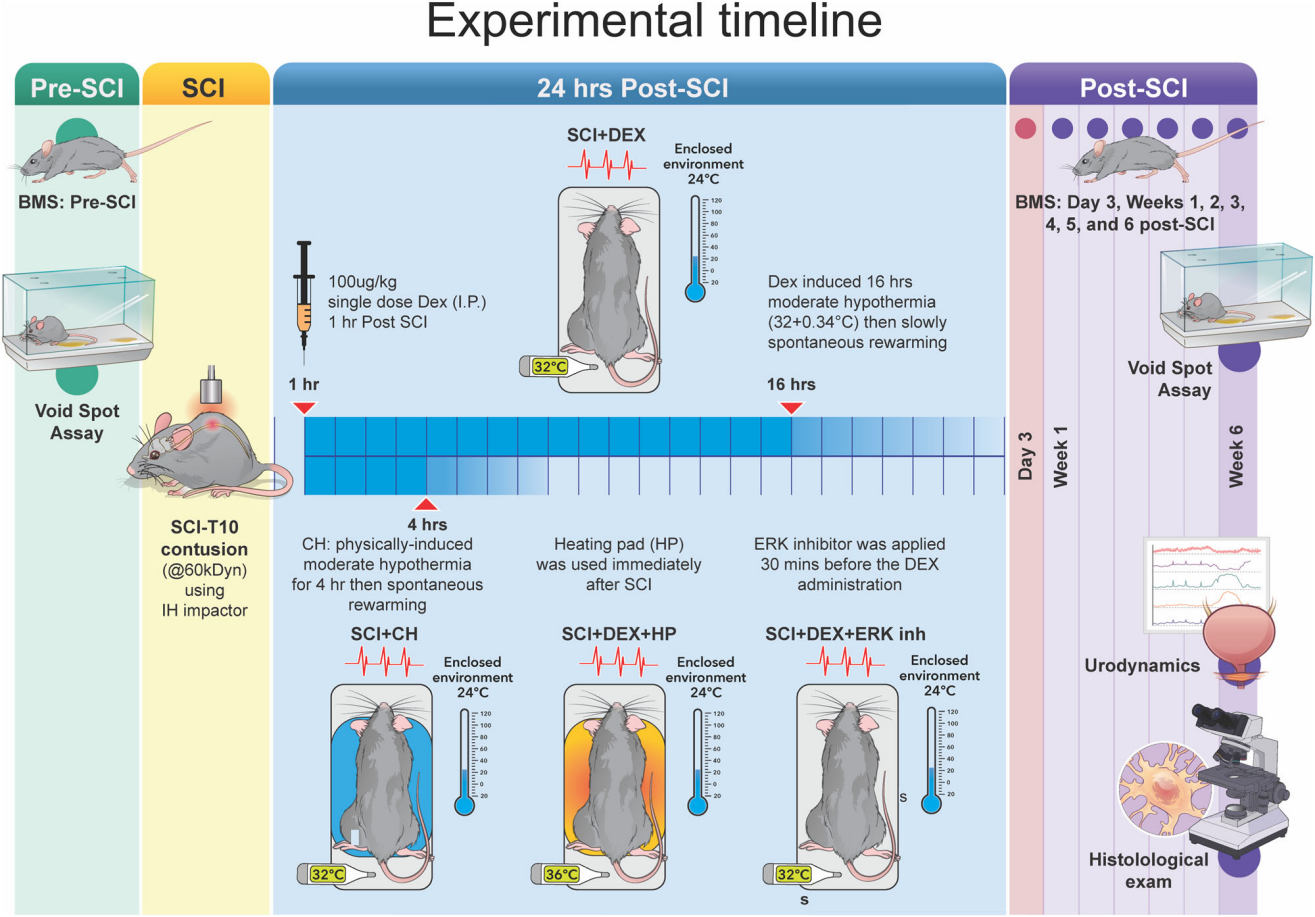
Experimental timeline of therapeutic efficacy of Dex-induced hypothermia after SCI. Schematic illustrating pharmacological interventions together with the cooling protocol, showing the duration of hypothermia and subsequent rewarming. The timeline also indicates scheduled evaluations of locomotor recovery, bladder function, and histological endpoints from acute (3, 24 h) through subacute (3–14 d) intervals up to 6 weeks post-SCI. SCI, spinal cord injury; Dex, dexmedetomidine; CH, conventional hypothermia; HP, heating pad; ERK, extracellular signal-regulated kinase; Inh, inhibitor; BMS, basso mouse scale; i.p., intraperitoneal; h, hour.

Animals were randomly assigned to three prespecified cohorts (Fig. 1). Animals were randomly assigned to treatment and control groups using computer-generated randomization. Allocation was performed after baseline assessments and before surgery. Confounders were reduced by randomizing testing order, rotating cage location, and performing all measurements at fixed times of day. Allocation was performed by an investigator separate from data collection. Experimenters remained blinded during surgeries, behavioral testing, outcome scoring, and data analysis. The functional-outcome cohort (male and female; *n* = 10/sex/group) included groups comprising Sham, SCI, SCI with Dex-induced pharmacologic hypothermia (SCI + Dex), and SCI with conventional physical hypothermia (SCI + CH). The mechanistic cohort (male; *n* = 10/group) included Sham, SCI, SCI + Dex, SCI with ERK pathway inhibition (SCI + Inh), SCI + Dex with active warming (heating pad; HP) to prevent hypothermia (SCI + Dex + HP), and SCI + Dex combined with ERK Inh (SCI + Dex + Inh). In both cohorts, animals were followed for 6 weeks postinjury for functional, behavioral, and histological assessments. A molecular cohort (male; *n* = 5/group/time point) was harvested at 3 h, 24 h, 3 d, 7 d, and 14 d postinjury to quantify molecular and inflammatory readouts in the spinal cord tissue.

All procedures, including behavioral testing, tissue processing, imaging, and data analysis, were performed under double-blind conditions. Animals were excluded a priori if their 24 h BMS score exceeded 0.5, if impact force deviated by >2 SD from the cohort mean or registered >10% error, or if they developed severe complications or died before the required survival period.

### Temperature control and pharmacological interventions

All surgeries were performed on a heating pad (37.0 ± 0.5°C). For SCI + Dex, mice were moved postoperatively to a Thermacage (Braintree Scientific; 24.0 ± 0.5°C) and given Dex (100 µg/kg, i.p.) 1 h after injury to induce moderate hypothermia (32.0 ± 0.5°C); animals remained in the cage until spontaneous rewarming. In SCI + Dex + HP, the same Dex regimen was used, but normothermia was maintained with a heating pad to prevent cooling. SCI + CH animals received surface cooling to 32.0 ± 0.5°C for 4 h—the duration shown to optimize functional recovery (Wang and Pearse, 2015; Yousefifard et al., 2020)—followed by spontaneous rewarming. SCI + Inh mice received the ERK inhibitor LY3214996 (50 mg/kg, i.p.) 30 min after injury, whereas SCI + Dex + Inh mice received the inhibitor 30 min before Dex at the same doses and routes (Fig. 1).

To model a realistic early-treatment window, Dex was administered 1 h postinjury, a clinically plausible timing given trauma-system targets for “golden hour” care (Okada et al., 2020; Newgard et al., 2022) and typical US prehospital intervals of ∼40–60 min for acute SCI (Newgard et al., 2015; Kreinest et al., 2017). Temperature metrics were computed per mouse and averaged, including nadir temperature, time to nadir, duration <32°C, and rewarming slopes. The net rewarming slope was defined as the linear rate of core temperature rise (degrees Celsius per hour) from the first sustained postnadir increase to return to normothermia.

### Vital signs and ECG acquisition

Physiological variables were recorded using a rodent monitoring system (HPMS, Harvard Apparatus). Measurements were taken at baseline, immediately after injury, 30 min after Dex or vehicle, hourly to 6 h, and at 24 h under light isoflurane. Core temperature was measured rectally; heart rate, respiratory rate, and arterial oxygen saturation were obtained from platform sensors and a hindlimb pulse-oximeter clip. Electrocardiograms were collected supine using surface limb leads (American Heart Association configuration), acquiring up to 120 s at 1,000 Hz (50–300 Hz bandpass). Artifact-free segments were used to identify P waves, QRS complexes, and J points, and PP, PR, and RR intervals were averaged over ≥15 beats (Oestereicher et al., 2023). Data were batch-processed in Clampfit v11.4.1 under blinded conditions.

Dex safety analyses (*n* = 5 per time point) were performed per mouse. Absolute values were recorded at each time point, and percentage change was calculated relative to each animal's pre-SCI baseline. Group data are reported as mean ± SEM. Beat-to-beat variability was quantified as the coefficient of variation (100 × SD/mean) and summarized as mean ± SEM across mice.

### Locomotion testing

Hindlimb locomotor recovery was evaluated using the nine-point Basso Mouse Scale (BMS) at baseline, Day 3, and weekly for 6 weeks, which assesses joint movement, plantar stepping, weight support, interlimb coordination, paw placement, trunk stability, and tail position. Scores range from 0 (complete paralysis) to 9 (normal locomotion). Two trained observers, blinded to the groups, averaged left and right hindlimb scores per mouse in an open field, according to established criteria.

### Void spot assay

At 6 weeks postinjury, spontaneous voiding was assessed using the void spot assay (Hill et al., 2018). After bladder expression and saline loading, mice were singly housed on absorbent paper for 4 h; urine spots were imaged under UV on an iBright system and analyzed for count, area, size, and spatial distribution with Image J and Void Whizzard (Hill et al., 2018). The void spot assay is a well-established behavioral method to assess voluntary micturition patterns in rodents, integrating both bladder function and supraspinal control. In Sham mice, voluntary urination is spatially organized and typically characterized by large urine spots deposited in the cage corners, reflecting intact bladder capacity, coordinated sphincter relaxation, and normal higher-center control of voiding behavior. Specifically, total urine spot number reflects voiding frequency. An increased number indicates bladder hyperreflexia and/or impaired urine storage. Large urine spots (>3 cm diameter) represent coordinated voluntary voiding with sufficient bladder filling, whereas small urine spots (<3 cm) indicate incomplete voiding or leakage due to impaired bladder–sphincter coordination. Urine volume in the cage corners reflects normal, socially patterned, voluntary urination. Urine volume in the cage center reflects disorganized or involuntary voiding behavior commonly observed after SCI. Although multiple parameters were analyzed, each captures a distinct aspect of voiding behavior (frequency, volume per void, and spatial organization), which cannot be inferred from a single metric alone. Voided volume (VV) was estimated from a calibration curve generated by applying known saline volumes to identical papers and imaging under the same conditions. Immediately after the 4 h session, residual bladder urine was collected by manual expression onto a fresh filter paper, imaged and quantified identically to obtain residual volume (RV). Voiding efficiency (VE) was calculated as VE = [voided volume (VV)/(VV + RV)] × 100. VE reflects the overall effectiveness of the coordinated micturition reflex, integrating both: detrusor muscle contractile strength and urethral sphincter relaxation/outlet resistance.

Principal component analysis (PCA) was performed on standardized urine spot assay (VSA) parameters to compare overall urination patterns between SCI and treatment groups. Rather than focusing on individual voiding metrics, PCA was used to integrate multiple correlated measures—voiding frequency, urine spot size, and spatial distribution—into a reduced set of components representing composite urination phenotypes. This approach is biologically motivated by the fact that micturition is controlled by an integrated neural network and that SCI disrupts multiple aspects of urinary function simultaneously. PCA therefore enables assessment of whether treatment induces a coordinated shift in urination behavior toward a pattern resembling organized voluntary voiding, as observed in Sham animals.

“Awake cystometrogram (CMG) and EUS electromyography (EMG)” were performed 6 weeks postinjury following established methods (Khabbaz et al., 2024). Under isoflurane, a suprapubic PE-30 catheter was placed in the bladder and bipolar electrodes in the EUS. After ∼40 min recovery in prone position over a heating pad, mice received 0.9% NaCl at 0.01 ml/min for 40 min, while intravesical pressure (IVP) and simultaneous EUS-EMG were recorded continuously (WinDaq) and analyzed offline under blinded conditions (Clampfit v11.4.1).

### Quantitative urodynamics

Urodynamic recording provides a mechanistic, physiological assessment of lower urinary tract function that complements behavioral urine spot assays. Each parameter reflects a distinct component of bladder–sphincter coordination, which is known to be disrupted after SCI. Specifically, voids were identified by IVP rises, with amplitude (VA), contraction duration (CD), voiding interval (VI), frequency, and AUC_IVP measured. IVP-based voiding parameters reflect detrusor contractile strength, VE, and bladder storage capacity; nonvoiding contractions (NVCs) were transient IVP rises >8 cmH_2_O without voiding. NVCs reflect detrusor overactivity during the storage phase, indicating abnormal bladder reflexes in the absence of coordinated sphincter relaxation. EUS-EMG bursting patterns provide a direct readout of sphincter coordination during voiding. EUS-EMG bursting was segmented into active and silent phases, with silent-period duration recorded. First-void latency was defined as the time from the start of bladder infusion to the initial void. This parameter reflects bladder sensory function and central voiding threshold. Metrics were uniformly applied and correlations visualized by heat map, which minimized analytical bias and ensured that observed correlations reflected biological coupling of voiding parameters rather than methodological variability.

### Tissue processing and analysis

At 6 weeks, mice were perfused with PBS/4% PFA; spinal cords and bladders were postfixed, cryoprotected, sectioned (20–25 µm), and bladders weighed. Cresyl violet/eosin (Millipore Sigma) stained T9–T12 cord sections. Immunohistochemistry for NeuN (chicken, 1:500, Invitrogen; rabbit, 1:500, Invitrogen), SMI31(rabbit, 1:500, Millipore), CD68 (macrophages; rabbit, 1:500, Cell Signaling Technology), 5-HT(serotonergic fibers; rabbit, 1:500, Sigma-Aldrich), and c-Fos (neuron activity, mouse, 1:200, Abcam) was performed on spinal cord (T9–T12, L3–L6) and brainstem sections with Alexa Fluor secondaries (1:500) and DAPI; images were acquired at 10/20×, and signals/cell counts quantified in ImageJ/FIJI, normalized to Sham. Bladder Masson's trichrome (ab150686, Abcam) stained collagen (blue) and muscle (red). Western blots of the T11 tissue at 3 h–14 d postinjury probed p-ERK1/2, ERK1/2, and RIPK1, quantified relative to β-tubulin. Multiplex cytokine assays on T11 homogenates measured inflammatory mediators at the same time points, with all analyses performed in duplicate.

### Statistics

Sample sizes were determined a priori by power calculations based on expected effect sizes from pilot data and the literature, using *α* = 0.05 and power (1 − *β*) = 0.8. Calculations targeted the primary outcome (BMS for locomotion or VE for bladder), accounted for the planned statistical test (e.g., two-way repeated–measure ANOVA or one-way ANOVA), and were inflated to allow for attrition (≈10–20%). Group sizes thus balance statistical sensitivity with the principle of using the minimum number of animals necessary. Analyses were blinded, using animals as the unit; results are mean ± SEM (*α* = 0.05). Normality and variance were assessed by Shapiro–Wilk and Levene tests. Parametric data were analyzed using one- or two-way ANOVA with Tukey or Welch/Games–Howell tests; nonparametric data were analyzed using the Mann–Whitney or Kruskal–Wallis test with Dunn test. Repeated measures were analyzed using two-way RM-ANOVA or mixed-effects models. Correlations were assessed using Pearson's or partial Spearman’s correlation coefficients; PCA was performed on *z*-scored variables with 90% confidence ellipses. Multiplex and Western blots were analyzed two-way ANOVA with Benjamini–Hochberg FDR (*q* < 0.05). Linear regression estimated slopes; effect sizes (*η*², *d*) were reported. Analyses were conducted using GraphPad Prism v10 and Python 3.11–3.13 (statsmodels, SciPy, scikit-learn).

#### Data availability

Data will be publicly released via publications and, upon acceptance, uploaded to PubMed Central. Datasets, including those in ODC-SCI, will be accessible through public quarriable repositories in a timely manner.

## Results

### Dex induces dose-dependent hypothermia in naive mice

**Figure 2.**
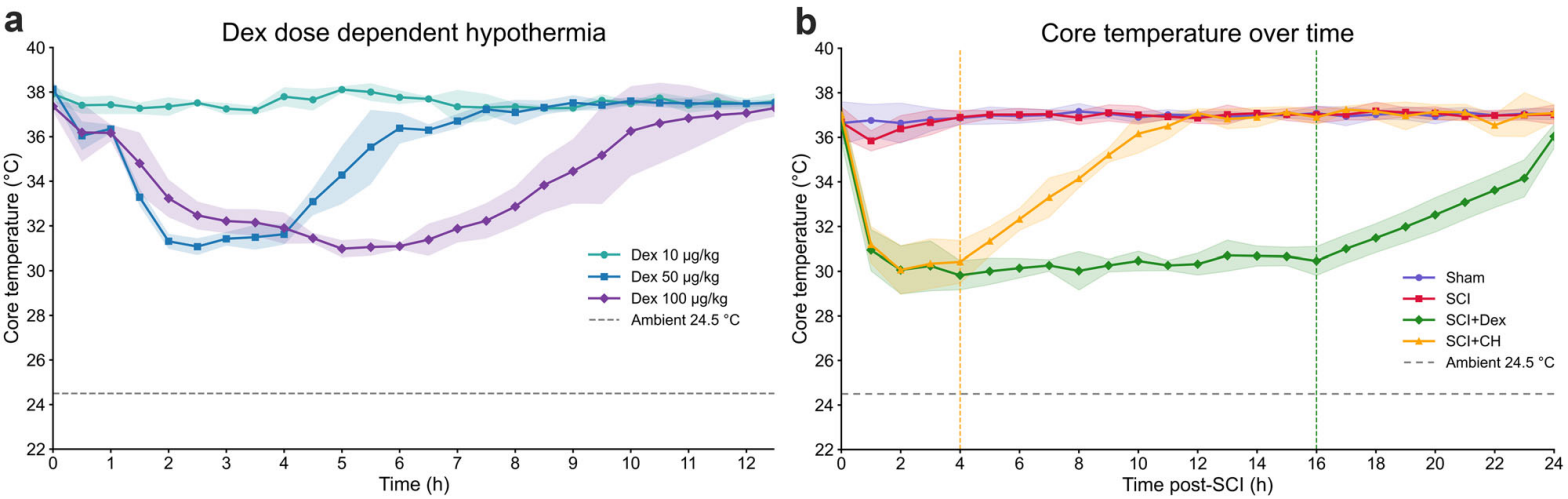
Dex induces dose-dependent hypothermia and distinct rewarming kinetics compared with conventional hypothermia. ***a***, Core temperature time course in naive mice (24.5°C) after Dex at 10, 50, or 100 µg/kg: 10 µg/kg showed no cooling, 50 µg/kg showed transient moderate hypothermia, and 100 µg/kg showed sustained moderate hypothermia. ***b***, Post-SCI core temperature over 24 h at 24.5°C: 100 µg/kg Dex at 1 h postinjury induced ∼16 h moderate hypothermia with gradual rewarming (∼0.23°C/h, onset at ∼16 h, green dashed line). CH reached a similar nadir but rewarmed faster (∼0.91°C/h, onset at ∼4 h, yellow dashed line). Data, mean ± 95% CI; *n* = 5/group. Two-way ANOVA for temperature trajectories; one-way ANOVA with Tukey post hoc for nadir and rewarming metrics. SCI, spinal cord injury; Dex, dexmedetomidine; CH, conventional hypothermia.

Naive C57BL/6 mice received Dex (10, 50, 100 µg/kg, i.p.) at 24.5°C. Only 100 µg/kg produced consistent, sustained moderate hypothermia with slow rewarming (full recovery 10.4 ± 0.53 h); 50 µg/kg caused brief, reversible cooling, and 10 µg/kg had no effect (Fig. 2*a*). A 200 µg/kg pilot was lethal. Based on these results and prior evidence of neuroprotection in TBI models (Wu et al., 2018), 100 µg/kg was chosen for subsequent SCI studies.

### Dex and conventional cooling/hypothermia (CH) induce hypothermia with distinct thermoregulatory profiles after SCI

At 24.5°C, a single 100 µg/kg Dex dose 1 h post-SCI induced sustained moderate hypothermia (∼29°C by 3.4 h), maintained at <32°C for >18 h with slow, controlled rewarming (∼0.23°C/h; Fig. 2*b*, green dashed line). In contrast, SCI + CH hypothermia lasted ∼5 h with faster rewarming (∼0.91°C/h; Fig. 2*b*, yellow dashed line). Dex provided a longer, more stable hypothermic window than conventional cooling, aligning with recommended pacing for therapeutic hypothermia (Omairi and Pandey, 2025), enabling effective early SCI intervention without external devices.

### Physiological safety of Dex-mediated hypothermia over 24 h assessed by vital signs and ECG

Because α_2_-agonists can cause bradycardia (Lewis et al., 2022) and hypothermia may predispose to arrhythmias (Polderman, 2009), vital signs and ECG were monitored for 24 h after Dex-induced hypothermia post-SCI (Supplemental Fig. 1). Dex caused transient HR and RR reductions, consistent with known pharmacology (Weerink et al., 2017), recovering by 24 h, with stable oxygen saturation. ECG showed early PP/RR changes but no PR prolongation, suggesting no atrioventricular conduction delay; beat-to-beat variability remained low, supporting stable sinus rhythm during recovery. Overall, 100 µg/kg Dex induced target hypothermia while preserving cardiorespiratory and rhythm stability, with full recovery and no mortality, supporting its acute physiological safety (Omairi and Pandey, 2025).

### Dex-induced hypothermia outperforms conventional cooling in promoting locomotor and voiding functional recovery after SCI

**Figure 3.**
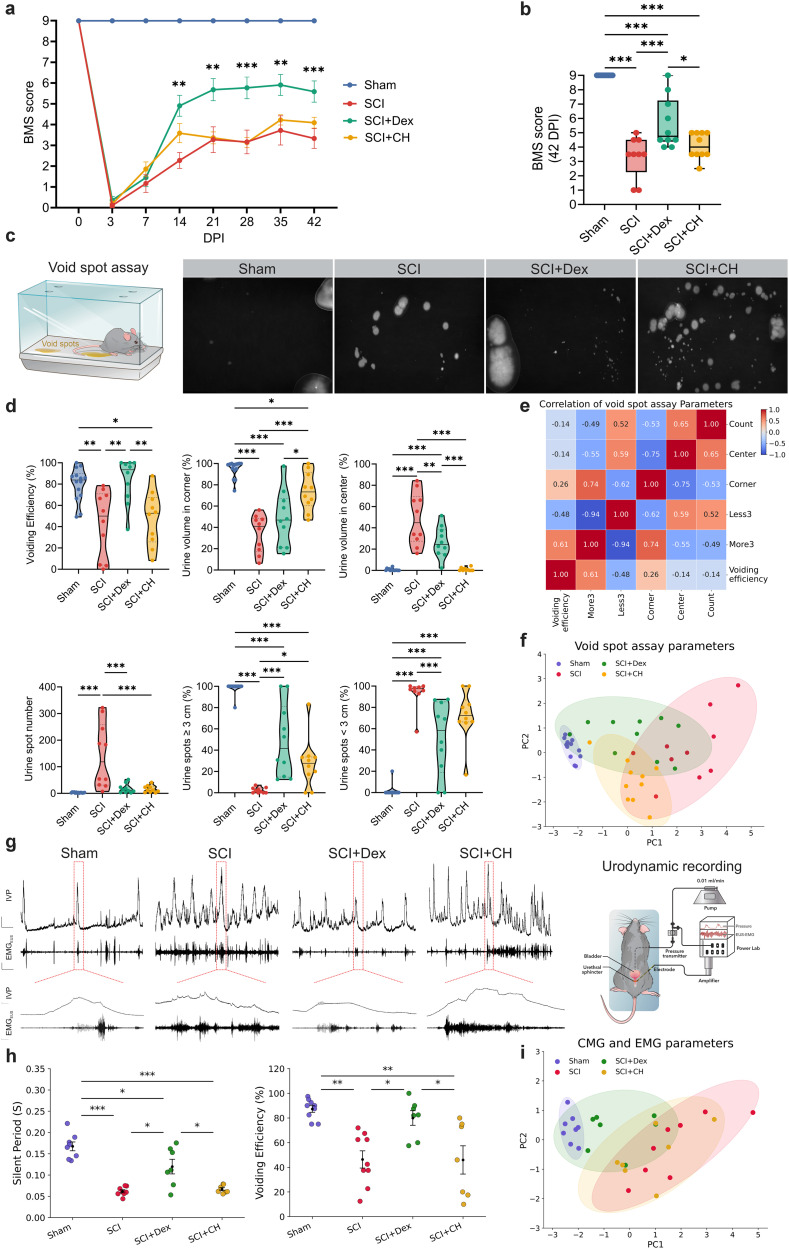
Dex improves locomotor recovery and bladder voiding 6 weeks after SCI in male mice. ***a***, BMS scores from 3 to 42 dpi (*n* = 10/group) showing progressive recovery. ***b***, Endpoint BMS at 42 dpi. ***c***, VSA schematic and representative UV images illustrating injury-associated small central spots and treatment-associated larger peripheral spots. ***d***, Violin plots of VSA metrics (spot count, spatial distribution, spot-size fractions, VE). ***e***, Correlation heatmap linking physiological voiding features (corner localization, larger spots) with higher VE. ***f***, PCA of VSA metrics (90% ellipses) separating groups by void quality and efficiency. ***g***, Representative CMG and simultaneous EUS-EMG traces with schematic. Top, Continuous recordings highlighting increased NVCs after SCI (scale bars, IVP 4 cm H_2_O; EUS 1 mV; time, 1 min). Bottom, Expanded void-phase traces showing coupling of IVP oscillations with EUS activity (scale bars, IVP 1 cm H_2_O; EUS 0.1 mV; time, 1 s). ***h***, Dot plots showing voids occur during extended sphincter SPs and associate with higher VE. ***i***, PCA of CMG and EUS-EMG variables (90% ellipses); higher PC1 reflects lower VE, shorter/absent SPs, and disordered pressure dynamics (injury-like phenotype). Time course data were analyzed by two-way ANOVA with Tukey post hoc tests; single-time-point comparisons by one-way ANOVA with Tukey. VSA distributions shown as medians with IQR. Data are mean ± SEM. *p < 0.05; **p < 0.01; ***p < 0.001. SCI, spinal cord injury; Dex, dexmedetomidine; CH, conventional hypothermia; BMS, Basso Mouse Scale; dpi, days postinjury; EUS-EMG, external urethral sphincter EMG; IVP, intravesical pressure.

Hindlimb locomotion (BMS) was tracked for 42 dpi. All groups were similarly impaired initially, but recovery diverged midcourse: at 3 dpi there were no between-group differences (male, Dex 0.36 ± 0.18 vs SCI 0.11 ± 0.11; *p* = 0.54; vs CH 0.18 ± 0.14; *p* = 0.74; female, Dex 1.65 ± 0.86 vs SCI 0.20 ± 0.11; *p* = 0.38; vs CH 0.40 ± 0.15; *p* = 0.50). By midrecovery, the trajectories diverged. In males, Dex climbed steadily and peaked at 35 dpi (5.91 ± 0.50), exceeding SCI (*p* < 0.01) and CH (*p* = 0.03); CH improved but plateaued lower (peak 4.23 ± 0.25 at 35 dpi). At 42 dpi, Dex remained higher (5.60 ± 0.51 vs CH 4.09 ± 0.26; *p* = 0.02). Females showed the same pattern, reaching 6.40 ± 0.65 at 35 dpi (*p* = 0.03 vs SCI; *p* < 0.01 vs CH) and remaining elevated at 42 dpi (6.25 ± 0.64 vs CH 3.55 ± 0.45; *p* < 0.01). Together, Dex-treated mice showed significantly greater and sustained functional improvement than SCI and CH groups in both sexes (Fig. 3*a*,*b*; Supplemental Fig. 2).

To evaluate bladder function in the same cohort, spontaneous voiding was assessed at 42 dpi across all experimental groups. Representative ultraviolet images of filter papers showed clear group differences. SCI produced numerous small, centrally distributed spots consistent with incontinence, whereas both interventions shifted voiding toward the periphery with fewer and larger spots (Fig. 3*c*). Numeric summaries mirrored these patterns. Total spot count was high in SCI at 118.5 [31–259.3] and lower with both treatments, Dex at 12 [4.5–24.5] with *p* < 0.001 versus SCI and CH at 11.5 [3.8–27.8] with *p* < 0.001 versus SCI, while Sham was 3 [1–4]. Spot-size distributions also shifted. SCI was dominated by small voids <3 cm at 97.51% [94.52–100], while the proportion of larger voids >3 cm increased with treatment, Dex at 41.7% [14.8–81.25] and CH at 27.72% [13.04–33.62]. Spatially, voids redistributed toward the corners in both groups, CH at 73.40% [60.41–91.36] and Dex at 46.96% [21.1–61.8], whereas SCI remained relatively centralized with the center at 44.76% [27.44–69.4]. Most importantly, VE improved with Dex to 80.31% [59.12–99.25], higher than CH at 52.65% [25.71–67.40] with *p* < 0.01 and SCI at 49.98% [9.42–73.73] with *p* < 0.01, whereas CH mainly reduced the spot number without fully normalizing efficiency. Because distributions were skewed and bounded with unequal variances, we report medians with interquartile ranges in Figure 3*d* and provide means with SEM in Supplemental Figure 3*a*. Correlative structure supported these patterns. Metrics indexing physiologic voiding, including greater corner localization and a higher fraction of spots >3 cm, were negatively correlated with fragmented features such as numerous spots <3 cm and centralized urination, and VE tracked with the physiological cluster (Fig. 3*e*). PCA summarized these relationships in two dimensions, with PC1 at 61.6% and PC2 at 18.9% of the variance. SCI separated from Sham along PC1, reflecting many small, central spots, and both treatments shifted toward Sham, with SCI + Dex clustering closer than SCI + CH, particularly along PC2, consistent with superior restoration of bladder emptying in the Dex group (Fig. 3*f*).

Because the VSA do not show detrusor pressure or sphincter coordination, we also performed awake CMG with concurrent EUS-EMG as a complementary physiological readout. We next examined bladder–sphincter physiology in the functional cohort. Representative traces from male mice showed SCI disrupting detrusor–sphincter coupling, with weak bursting and inefficient voiding. Both treatments partially restored patterning, and Dex most closely resembled Sham, with EUS bursting consisting of alternating active periods (APs) and silent periods (SPs) that coincided with rapid IVP oscillations in the CMG tracing (Fig. 3*g*). To capture the interdependence among these cystometric and sphincter variables, we generated a correlation heatmap (Supplementary Fig. 4), which revealed strong positive associations between physiological parameters such as VE, sphincter SP, and CD and inverse relationships with pathological features including frequent NVC and delayed first-void latency. This multivariate structure supported the use of PCA to integrate bladder function outcomes across groups. During voiding, time to first void and NVCs increased in SCI, consistent with detrusor–sphincter discoordination, but Dex reduced both toward Sham, whereas CH remained closer to SCI. Voiding amplitude (VA), CD, and voiding contractions per minute did not differ significantly among groups; Dex shifted these toward Sham, with mixed effects under CH (Table 1). VE was reduced by SCI (49.2%) relative to Sham (87.2%). Dex increased VE to 79.93%, significantly higher than SCI (49.22%) and CH (45.93%), which did not improve versus SCI (Fig. 3*h*). EUS activity with Dex revealed a longer SP of 0.12 s, whereas CH was shorter at 0.07 s and similar to SCI at 0.07 s (Fig. 3*h*). Accordingly, the VI was shorter in CH and longer in Dex, consistent with CH increasing void frequency without normalizing efficiency, whereas Dex emphasized coordinated, effective voids. These findings indicate that Dex improved both bladder emptying and sphincter coordination beyond surface cooling. PCA integrated these readouts. The first two components explained roughly 55 and 24% of the variance. PC1 captured a continuum of emptying efficiency, sphincter bursting coordination, and void quality, with top contributors including VE, SP, CD, VA, and time to first void. Sham scored highest on PC1 and SCI lowest. Dex shifted strongly toward Sham with minimal overlap with SCI, whereas CH showed only partial movement and overlapped SCI. Group 90% ellipses indicated smaller dispersion and a larger shift for Dex than for CH, consistent with a broader restoration of bladder–sphincter function (Fig. 3*i*).

**Table 1.** CMG and EUS-EMG parameters in male mice comparing Dex with conventional hypothermia

	Sham (*n* = 9)	SCI (*n* = 10)	SCI + Dex (*n* = 7)	SCI + CH (*n* = 7)
CMG parameters				
VA, cmH2O	14.13 ± 1.36	26.85 ± 2.08*	21.92 ± 3.05	24.1 ± 3.44*
CD, S	14.69 ± 0.82	22.83 ± 2.84*	19.3 ± 1.91	20.87 ± 2.68
VI, S	86.88 ± 13.24	122 ± 24.3	115.3 ± 16.6^$^	38.76 ± 8.01^#^
Voiding contraction, number/minute	0.91 ± 0.19	0.27 ± 0.05*	0.62 ± 0.19	0.39 ± 0.11
NVC, number/minute	0.26 ± 0.09	1.58 ± 0.28***	0.34 ± 0.09^###, $$$^	2.08 ± 0.22***
Area IVP, cmH2O.S	97.4 ± 7.47	279.5 ± 36.88***	206.65 ± 29.96	189.94 ± 23.49
Area IVP/bursting period, cmH2O	8.38 ± 0.99	43.74 ± 10.65*	5.68 ± 1.42^#, $$$^	73.09 ± 17.67***
First voiding, S	85.41 ± 14.4	461.4 ± 67.34***	204.45 ± 46.01^#^	365.69 ± 94.45*
VE, %	87.22 ± 2.71	49.22 ± 6.87**	79.93 ± 5.92^#, $^	45.93 ± 11.45**
EMG parameters during voiding bladder contraction				
Bursting period, S	3.27 ± 0.53	0.92 ± 0.10**	2.98 ± 0.26^#^	2.82 ± 0.84
SP, S	0.18 ± 0.02	0.07 ± 0.01***	0.12 ± 0.02^#,$^	0.07 ± 0.0

Values are mean ± SEM. Group comparisons used one-way ANOVA with Tukey's post hoc; if variance was unequal, Welch's ANOVA with Games–Howell. Symbols mark post hoc differences: * versus Sham, ^#^ versus SCI, ^$^ versus SCI + CH; one, two, and three symbols denote *p* < 0.05, *p* < 0.01, and *p* < 0.001, respectively.

CMG, cystometrogram; EUS-EMG, external urethral sphincter electromyography; IVP, intravesical pressure; SCI, spinal cord injury; Dex, dexmedetomidine; CH, conventional hypothermia.

### Dex preserves the spinal cord white and gray matter after SCI

**Figure 4.**
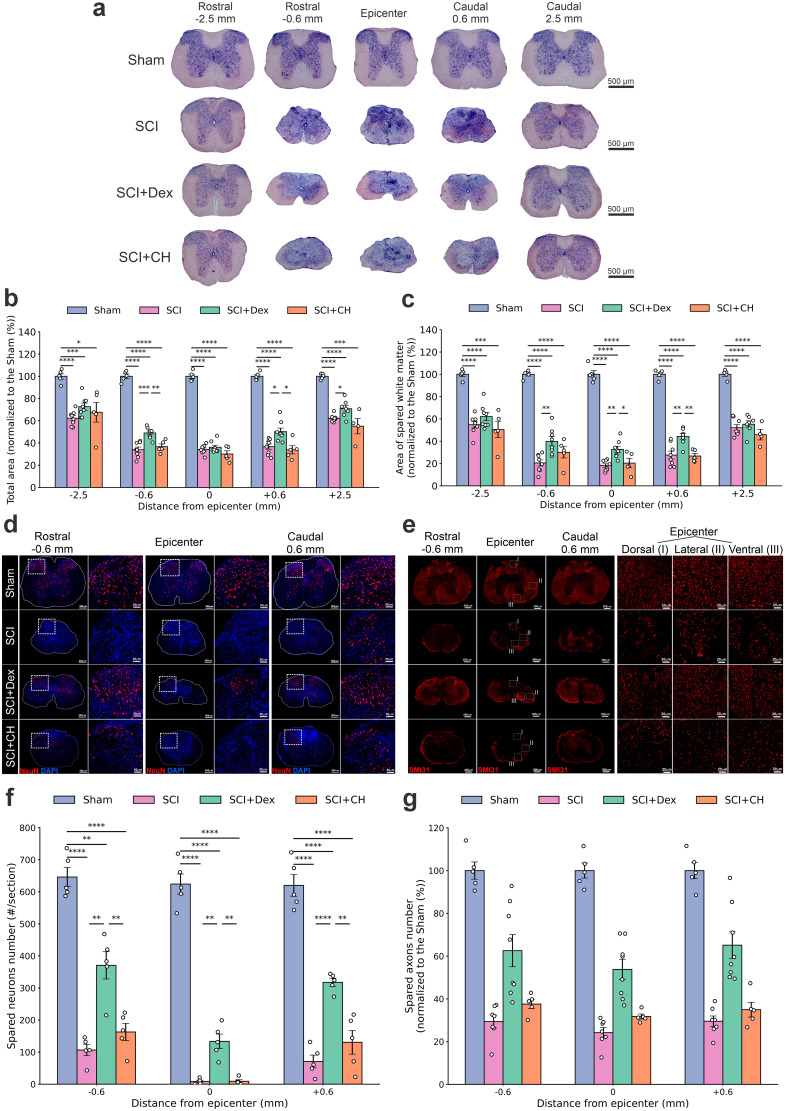
Dex preserves the spinal cord tissue, neurons, and axons after SCI. ***a***, Representative large-field cresyl violet and eosin images along the T9–T12 axis (epicenter; rostral −0.6, −2.5 mm; caudal +0.6, + 2.5 mm). Scale bars, 500 µm. ***b***, Total cross-sectional cord area (normalized to Sham). ***c***, WM area preservation (normalized to Sham). ***d***, Immunofluorescence for NeuN (neurons, red) and DAPI (nuclei, blue) at the epicenter and ±0.6 mm showing neuronal loss and sparing; right insets highlight surviving neurons (scale bars, main, 200 µm; inset, 50 µm). ***e***, SMI31 immunostaining (axons) at the epicenter and ±0.6 mm; insets (I–III) show dorsal, lateral, and ventral funiculi integrity (scale bars, main, 200 µm; insets, 20 µm). ***f***, NeuN-positive neuron counts per section across epicenter and perilesional segments. ***g***, Quantification of SMI31 signal indicating axonal sparing. Data are mean ± SEM; two-way ANOVA with Tukey post hoc. SCI, spinal cord injury; Dex, dexmedetomidine; CH, conventional hypothermia.

Histological analysis showed severe tissue loss after SCI, with ∼66% reduction at the epicenter and perilesional zones (±0.6 mm). Both Dex and CH improved preservation, but Dex was superior, especially perilesionally (−0.6 mm, Dex–CH Δ = 12.31%; *p* < 0.01; +0.6 mm, Δ = 16.34%; *p* < 0.05) and flattened the rostrocaudal loss profile (Fig. 4*a*,*b*). White matter (WM) loss was pronounced (Sham–SCI Δ = 81.87%); Dex preserved significantly more WM than CH at +0.6 mm (Δ = 17.55%; *p* < 0.01) and trended higher at the epicenter (*p* = 0.081; Fig. 4*c*). Dex also better maintained gray matter architecture. Overall, Dex-induced hypothermia provided superior white and gray matter protection, motivating NeuN and SMI31 analyses.

### Dex enhanced neuronal survival and axonal sparing after SCI

Gray matter architecture was preserved in Dex-treated cords, unlike the severe disruption in untreated injury. NeuN staining showed robust neuronal rescue at the epicenter (Δ = 125.0; *p* = 0.013) and perilesional zones (Δ = 264.2; *p* < 0.01), with Dex outperforming conventional cooling (epicenter Δ = 124.4; *p* = 0.013; perilesion Δ = 187.0; *p* = 0.010; Fig. 4*d*,*f*). SMI31 staining revealed parallel axonal protection, with increased sparing at the epicenter (Δ = 29.58%; *p* < 0.001) and adjacent regions (Δ = 33.19%; *p* = 0.008), again exceeding cooling (epicenter Δ = 22.04%; *p* = 0.004; perilesion Δ = 24.98%; *p* = 0.025; Fig. 4*e*,*g*). These results, together with improved total and WM preservation, indicate that Dex-induced hypothermia provides superior multicompartment structural protection consistent with its enhanced functional recovery.

### Dex preserved descending serotonergic input and raphe activation, consistent with improved bladder function

**Figure 5.**
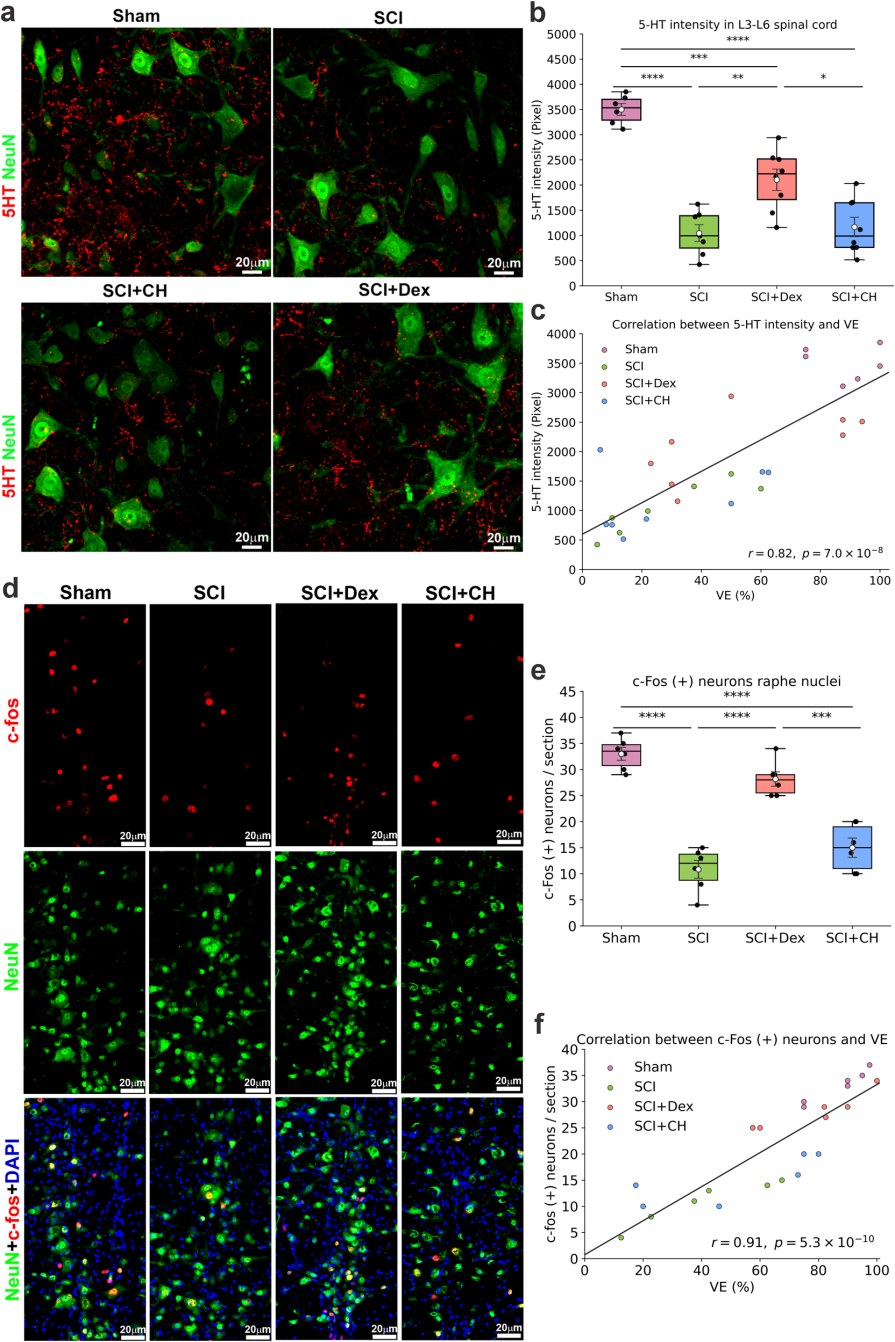
Dex preserves descending serotonergic input and raphe activation, aligning with improved voiding after SCI. ***a***, Immunofluorescence in lumbar ventral horn (L3–L6) showing 5-HT (red) and NeuN (green); scale bars, 20 µm. ***b*,** Box-and-whisker plots of 5-HT intensity; SCI reduced serotonergic input, Dex partially preserved it, and CH remained low. ***c***, Spearman correlation between 5-HT intensity and VE across animals. ***d***, Midline raphe images post-CMG showing c-Fos (red), NeuN (green), and DAPI (blue); scale bars, 20 µm. ***e***, Raphe c-Fos^+^ neuron counts: reduced after SCI, restored toward Sham with Dex, low with CH. ***f***, Correlation between raphe c-Fos^+^ counts and VE. Data are mean ± SEM; one-way ANOVA with Tukey post hoc (*p < 0.05 to ****p < 0.0001). SCI, spinal cord injury; Dex, dexmedetomidine; CH, conventional hypothermia; 5-HT, serotonin; VE, voiding efficiency.

SCI markedly reduced 5-HT signal in the lumbosacral cord, whereas Dex significantly preserved this input (*p* = 0.0054), outperforming conventional cooling (*p* = 0.011; Sham 3,498.53 ± 117.62; SCI 1,044.61 ± 166.82; Dex 2,104.10 ± 211.64; CH 1,167.43 ± 192.23; Fig. 5*a*,*b*). Higher 5-HT density strongly correlated with better VE (Fig. 5*c*, partial Spearman *r* = 0.572; *p* = 0.0012), indicating that Dex supports autonomic recovery by maintaining descending serotonergic control.

To assess functional engagement of spared serotonergic pathways (Ahn et al., 2018), c-Fos was quantified in the raphe nucleus after CMG (Fig. 5*d*). SCI markedly reduced activation, whereas Dex restored c-Fos expression (28.17 ± 1.38 vs 10.83 ± 1.70; *p* < 0.001), unlike conventional cooling, which showed little improvement (15.00 ± 1.84; *p* = 0.26 vs SCI; Fig. 5*e*). Raphe c-Fos strongly correlated with VE (*r* = 0.91; *p* = 5.3 × 10^−^¹⁰; Fig. 5*f*), indicating Dex preserves brainstem control of bladder function.

Chronic SCI caused bladder wall thickening and muscle loss, but Dex largely reversed these changes, restoring morphology near Sham levels, whereas conventional cooling produced only partial improvement (Supplementary Fig. 5*a*). Both treatments reduced bladder weight, but Dex achieved greater normalization, consistent with its superior voiding recovery (Supplementary Fig. 5*b*).

### Preventing hypothermia with a heating pad reduced Dex's therapeutic effects

To determine whether hypothermia is necessary for Dex-mediated therapeutic effects, we prevented Dex-induced hypothermia by maintaining male mice at normothermia on a heating pad (SCI + Dex + HP). We then compared locomotor and bladder recovery outcomes with Sham, SCI, and SCI + Dex to isolate the temperature-dependent component of Dex's effect.

**Figure 6.**
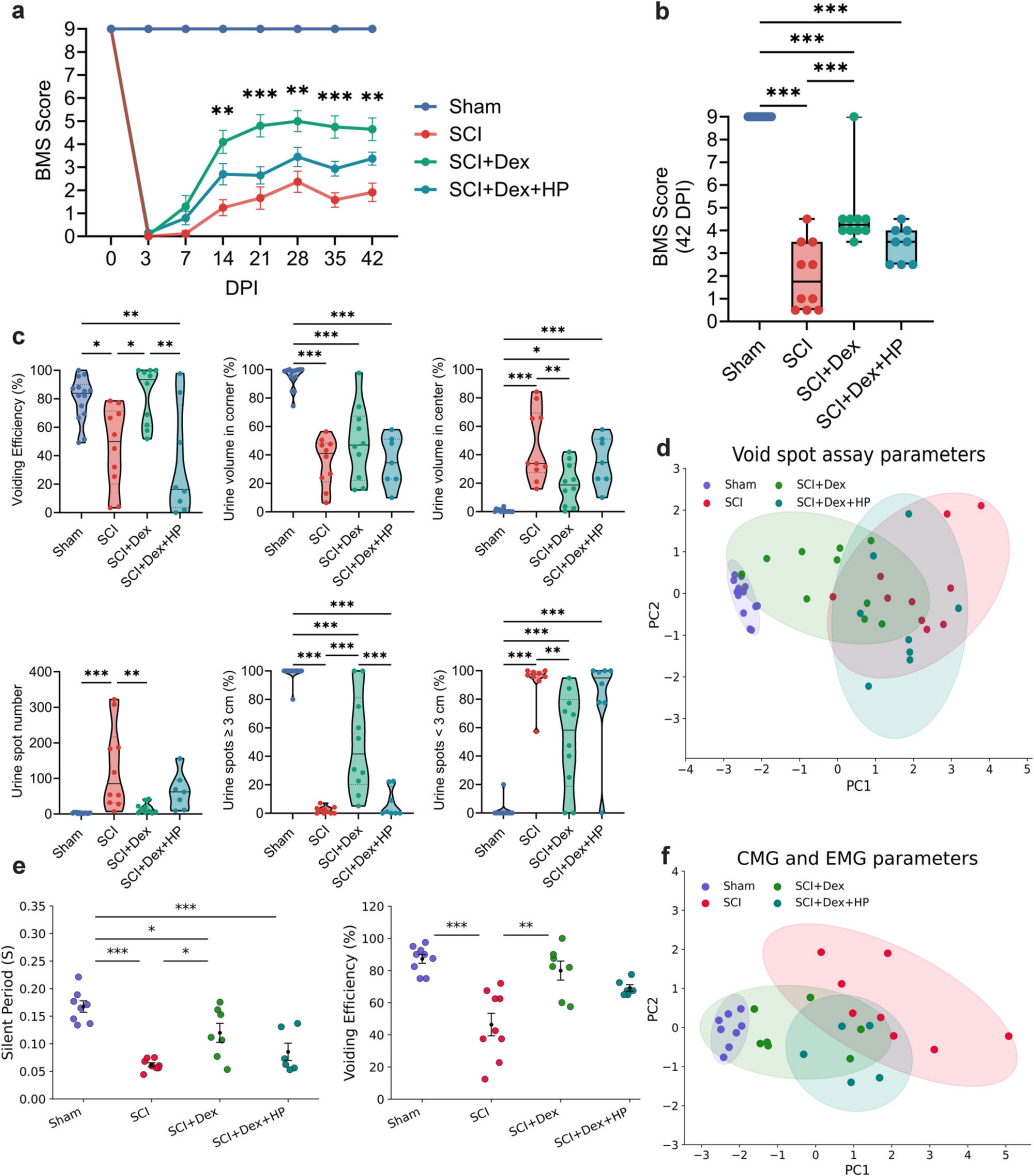
Active warming partially offsets Dex-induced functional recovery after SCI in male mice. ***a***, BMS time course from 3 to 42 dpi (*n* = 10/group). ***b***, Endpoint BMS at 42 dpi. ***c***, VSA violin plots showing spot count, spatial distribution, spot-size fractions, and VE across groups. ***d***, PCA (90% confidence ellipses) of VSA parameters summarizing multivariate voiding patterns. ***e***, Aligned dot plots of key urodynamic variables (sphincter SP, VE) showing warming partially diminishes Dex-associated gains. ***f***, PCA of CMG and EUS-EMG features (90% confidence ellipses) summarizing variance in emptying efficiency, sphincter timing, and pressure dynamics. Time courses: two-way ANOVA with Tukey post hoc; single-time-point, one-way ANOVA with Tukey; VSA distributions, median ± IQR; data, mean ± SEM. *p < 0.05; **p < 0.01; ***p < 0.001. SCI, spinal cord injury; Dex, dexmedetomidine; HP, heating pad; BMS, Basso Mouse Scale; DPI, days postinjury; CMG, cystometrogram; EUS-EMG, external urethral sphincter electromyography.

Hindlimb locomotion evaluation showed that at 3 and 7 dpi, both Dex and Dex + HP began to rise without between-group differences. By 14 dpi, SCI + Dex reached 4.10 ± 0.50 versus SCI 1.25 ± 0.35 (*p* < 0.001), then continued increase without a sustained drop, peaking near 28 dpi at 5.00 ± 0.45 and remaining 4.65 ± 0.50 at 42 dpi. The SCI + Dex + HP group also improved but consistently trailed Dex, with significant differences at 21 and 35 dpi (both *p* < 0.05; Fig. 6*a*). By 42 dpi, SCI + Dex + HP reached 3.38 ± 0.28 (*p* = 0.16 vs Dex; *p* = 0.036 vs SCI; Fig. 6*b*). Overall, preventing hypothermia partially reversed Dex's locomotor advantage, implicating cooling as a key contributor.

To evaluate Dex's effect on bladder function without hypothermia, the VSA showed that the spot number was high after SCI at 117 [32.0–243.0], fell with Dex to 19.0 [5.0–33.5], and remained elevated under active warming at 62.0 [11–95] (Sham 3.0 [1.0–4.0]). Spatial distribution followed the same direction. The corner area rose from 38.87% [19.2–47.6] in SCI to 40.41% [18.7–61.8] with Dex but was lower with Dex + HP at 34.5% [23.1–51.1] (Sham 98.61% [91.4–99.8]). Voiding in the center area decreased with Dex (21.29% [2.9–34.9]) vs SCI (33.9% [29.6–66.0]), with Dex + HP intermediate (34.55% [23.1–51.1]). Volume surrogates separated groups more clearly. The fraction of large voids rose with Dex to 28.57% [14.2–67.5] from 1.85% [0.00–4.2] in SCI but remained minimal with Dex + HP (0.53% [0.00–18.9]), while SCI was similarly low and Sham was large (100% [100–100]). Most importantly, VE improved with Dex to 80.31% [59.1–99.3], near Sham at 83.88% [68.8–90.1], but it was poor with Dex + HP at 16.37% [6.52–58.08] and variable in SCI (49.98% [9.4–73.7]). Thus, maintaining normothermia abrogated Dex's gains in large-void fraction and VE despite modest shifts in distribution and frequency. Medians with interquartile ranges are shown in Figure 6*c*, and means with SEM are shown in Supplementary Fig. 3*b*. PCA integrated these VSA metrics into two axes that explained 80.6% of the variance (PC1 64.7%, PC2 15.9%). PC1 tracked a pathological-to-physiological continuum with fewer small central spots and larger corner-localized spots, whereas PC2 reflected VE. Group centroids separate accordingly. Sham clustered at the physiological end, SCI clustered at the pathological end, SCI + Dex shifted strongly toward Sham, and SCI + Dex + HP clustered nearer SCI (Fig. 6*d*). Taken together, preventing hypothermia eliminated Dex's improvements in voiding behavior, consistent with a temperature-dependent mechanism (Fig. 6*d*).

Urodynamic measurements showed that HP attenuated Dex's bladder improvement effect. SCI + Dex + HP had lower VE (Dex 77.93 ± 4.82% vs Dex + HP 69.17 ± 2.01%), more NVC (*p* = 0.047), and a larger IVP area during voids (*p* = 0.001), indicating less efficient pressure–flow coupling when hypothermia was prevented. In the Dex group, SP duration was longer, reflecting prolonged sphincter relaxation (Fig. 6*e*), and first-void latency was shorter (*p* = 0.0012 and *p* = 0.004 vs SCI, respectively). These improvements were smaller in the Dex + HP. VA and CD did not differ significantly among groups and tended to fall between SCI and Sham for Dex, with Dex + HP intermediate. Both Dex and Dex + HP improved several readouts relative to SCI, but Dex consistently moved closer to Sham, whereas Dex + HP retained only part of the benefit (Table 2). These patterns support the conclusion that hypothermia is necessary for the full urodynamic effect of Dex.

**Table 2.** CMG and EUS-EMG parameters in male mice comparing Dex with active warming

	Sham (*n* = 9)	SCI (*n* = 10)	SCI + Dex (*n* = 7)	SCI + Dex + HP (*n* = 6)
CMG parameters				
VA, cmH2O	14.13 ± 1.36	25.79 ± 2.14**	21.91 ± 3.05	29.60 ± 3.19***
CD, S	14.69 ± 0.82	22.15 ± 2.63*	19.30 ± 1.91	22.70 ± 1.64
VI, S	86.88 ± 13.24	117.13 ± 22.26	143.08 ± 31.13	146.75 ± 74.44
Voiding contraction, number/minute	0.91 ± 0.19	0.31 ± 0.06*	0.62 ± 0.19	0.24 ± 0.08*
NVC, number/minute	0.26 ± 0.09	1.47 ± 0.28***	0.34 ± 0.09^##, $^	1.24 ± 0.24*
Area IVP, cmH2O.S	97.40 ± 7.47	266.65 ± 35.42***	206.65 ± 29.96^$$^	336.44 ± 35.60***
Area IVP/bursting period, cmH2O	8.38 ± 0.99	34.23 ± 10.02*^, #^	5.68 ± 1.42^##^	20.04 ± 10
First voiding, S	85.41 ± 14.40	466.19 ± 60.42***	204.45 ± 46.01^##^	273.28 ± 43.64^#^
VE, %	87.22 ± 2.71	49.22 ± 6.87***	77.93 ± 4.82^##^	69.17 ± 2.01
EMG parameters during voiding bladder contraction				
Bursting period, S	3.27 ± 0.53	0.92 ± 0.1***	2.98 ± 0.26^##^	2.29 ± 0.38
SP, S	0.17 ± 0.01	0.06 ± 0.00***	0.12 ± 0.02*^, #^	0.09 ± 0.02***

Values are mean ± SEM. Group comparisons used one-way ANOVA with Tukey's post hoc; if variance was unequal, Welch's ANOVA with Games–Howell. Symbols mark post hoc differences: * versus Sham, ^#^ versus SCI, ^$^ versus SCI + Dex + HP; one, two, and three symbols denote *p* < 0.05, *p* < 0.01, and *p* < 0.001, respectively.

CMG, cystometrogram; EUS-EMG, external urethral sphincter electromyography; IVP, intravesical pressure; SCI, spinal cord injury; Dex, dexmedetomidine; HP, heating pad.

To integrate correlated CMG and EUS-EMG variables, we performed PCA using the five top-loading features (VE, SP, CD, Area IVP, and first-void latency). The first two components explained 81.93% of variance (PC1 = 70.14%; PC2 = 11.79%). PC1 captured a continuum from efficient emptying with coordinated sphincter bursting to prolonged, inefficient pressure dynamics. Dex shifted strongly toward Sham, Dex + HP occupied an intermediate position short of Dex, and SCI remained at the pathological end (Fig. 6*f*). Together these findings indicate that maintaining normothermia blunts Dex's restorative impact on bladder emptying and sphincter coordination.

### Dex-induced hypothermia produced distinct time-dependent immunomodulation after SCI

**Figure 7.**
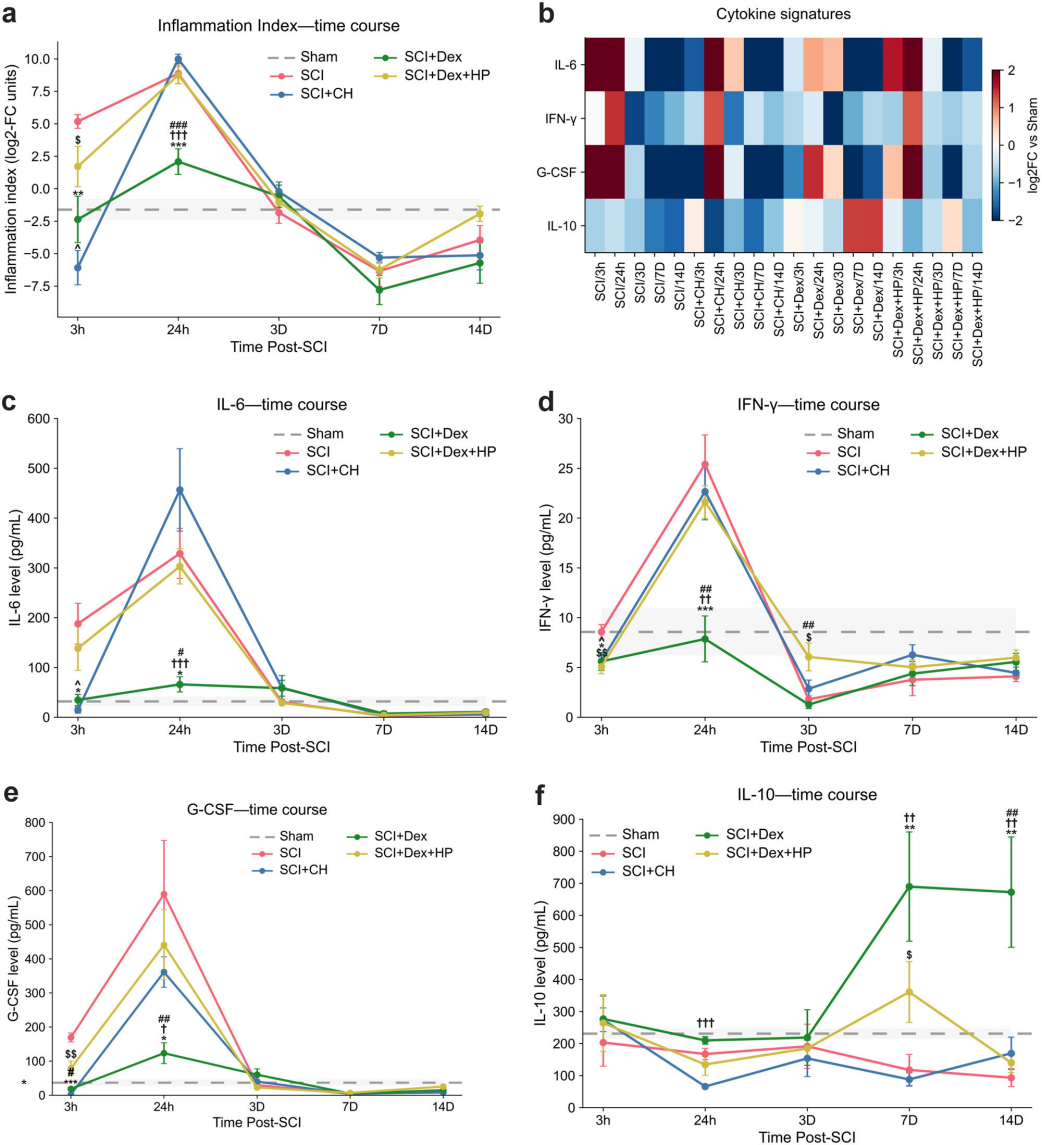
Anti- and proinflammatory cytokine profiles with Dex-induced hypothermia after SCI. ***a***, Composite inflammation index (log_2_ scale, relative to Sham) across acute and subacute time points; lower values indicate anti-inflammatory shift. Gray band, 95% CI. ***b***, Heatmap of cytokine modulation (IL-6, IFN-γ, G-CSF, IL-10) across groups and times; red, upregulation; blue, downregulation, log_2_FC relative to Sham. ***c–f***, Time courses of IL-6 (***c***), IFN-γ (***d***), G-CSF (***e***), and IL-10 (***f***). Data, mean ± SEM (*n* = 5 per group per time point; index *n* = 5–8). Composite index, one-way ANOVA; cytokines, two-way ANOVA with Tukey post hoc (*p < 0.05; **p < 0.01; ***p < 0.001). Symbols: * versus SCI; † versus SCI + CH; # versus SCI + Dex + HP; $ indicates SCI versus SCI + Dex + HP; ^ indicates SCI versus SCI + CH. SCI, spinal cord injury; Dex, dexmedetomidine; CH, conventional hypothermia; HP, heating pad.

Given that anti-inflammatory signaling is a mechanistically plausible route to neuroprotection for both hypothermia and α2-adrenergic agonism with Dex (Hu et al., 2022; Ransom et al., 2022), we tested their combined effects post-SCI. Multiplex cytokine analysis showed strong early suppression of proinflammatory mediators (IL-6, IFN-γ, G-CSF) at 24 h, greater than conventional cooling or Dex under normothermia (all *p* < 0.05 vs SCI and CH; *p* < 0.001 vs Dex + HP), followed by elevated IL-10 at 14 d (*p* < 0.01; Fig. 7*a*–*f*). This biphasic profile, supported by reduced macrophage accumulation at the lesion (Supplemental Fig. 6), indicates that Dex-induced hypothermia synergistically enhances anti-inflammatory and reparative signaling, outperforming hypothermia alone or Dex without cooling.

### ERK signaling is required for Dex-mediated neuroprotection and functional improvement after SCI

**Figure 8.**
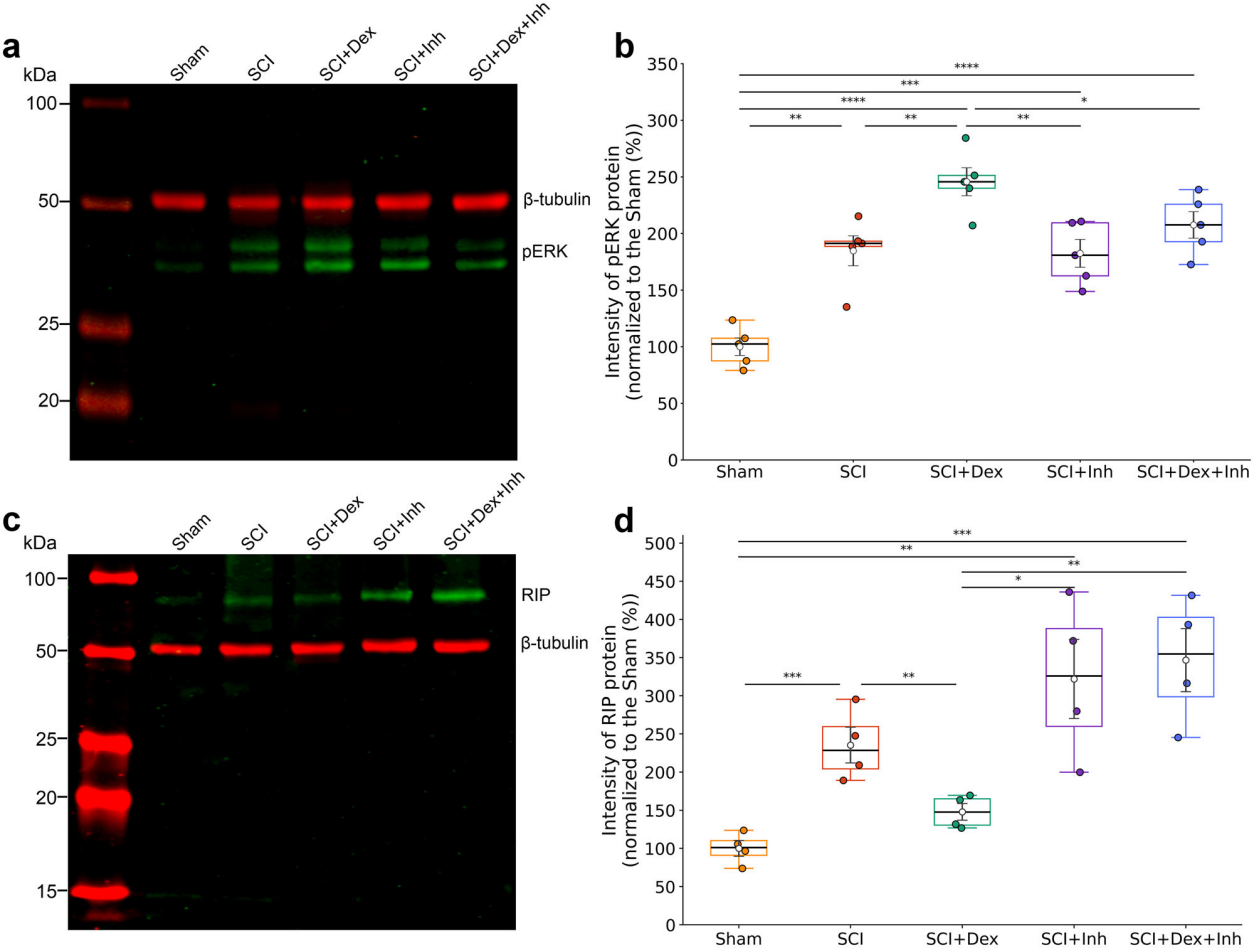
Dex modulates ERK and RIPK1 signaling after SCI. ***a***, Representative immunoblots at 3 h post-SCI showing pERK1/2 (∼42/44 kDa) with β-tubulin (∼50 kDa) as loading control. ***b***, Densitometric quantification of pERK1/2 at 3 h, normalized to β-tubulin and expressed relative to Sham, showing early Dex-induced ERK activation. ***c***, Representative immunoblots at 7 d post-SCI showing RIPK1 (∼70 kDa) with β-tubulin. ***d***, Quantification of RIPK1 at 7 d, normalized to β-tubulin and relative to Sham, showing Dex-associated reduction in necroptotic signaling. Data are mean ± SEM; one-way ANOVA with Tukey post hoc (*p < 0.05 to ****p < 0.0001). SCI, spinal cord injury; Dex, dexmedetomidine; Inh, ERK inhibitor; pERK, phosphorylate extracellular signal-regulated kinase; RIPK1, receptor-interacting protein kinase 1.

**Figure 9.**
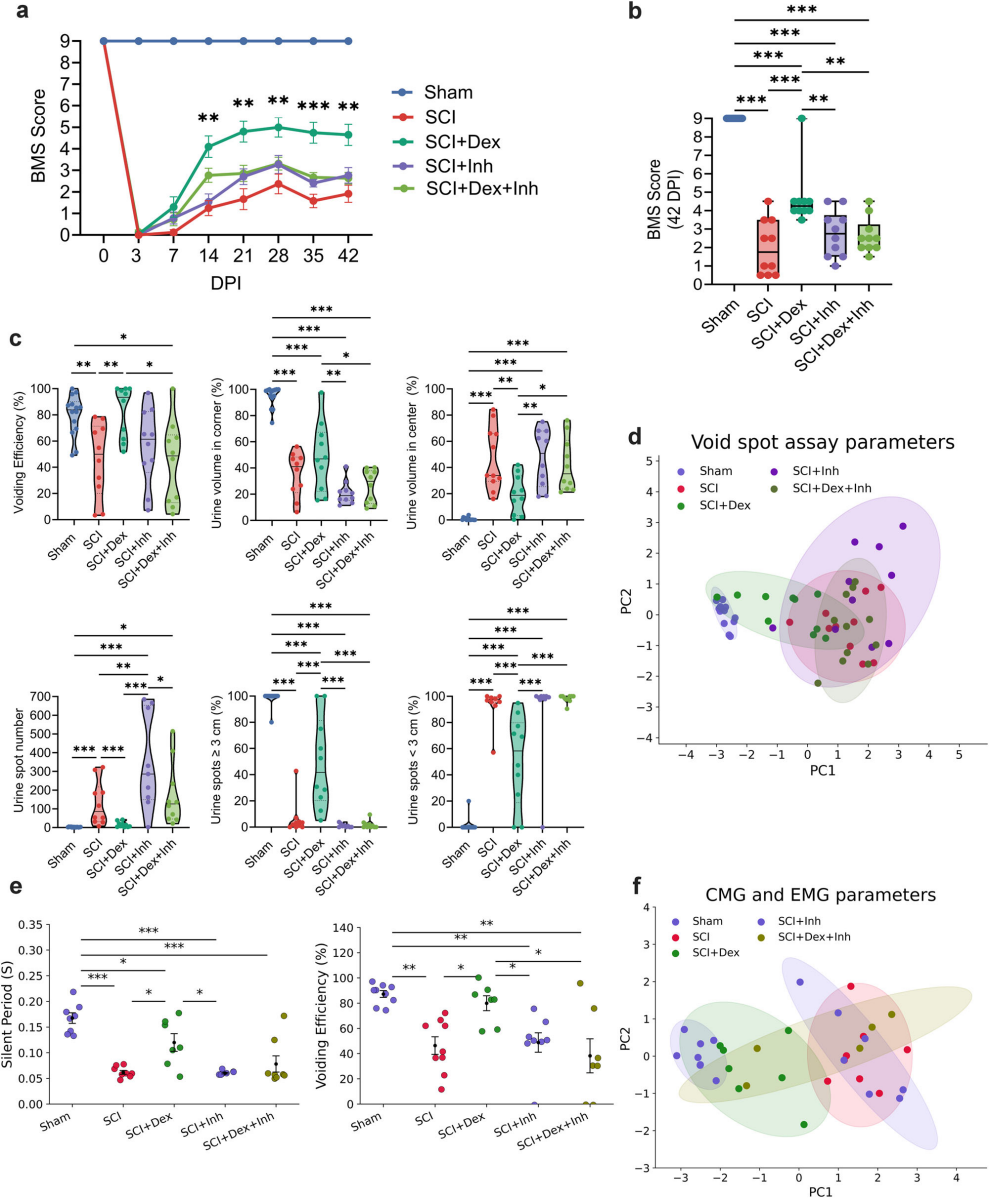
ERK Inh partially reverses Dex-induced functional recovery at 6 weeks post-SCI in male mice. ***a***, BMS trajectories from 3 to 42 dpi (*n* = 10/group). ***b***, Endpoint BMS at 42 dpi. ***c***, VSA violin plots showing spot count, spatial deposition, spot-size fractions, and VE. ***d***, PCA of VSA features (90% confidence ellipses) illustrating fragmentation versus organized voiding. ***e***, Aligned dot plots of urodynamic indices (sphincter SP, VE). ***f***, PCA of CMG and EUS-EMG features (90% confidence ellipses) summarizing bladder emptying, sphincter timing, and pressure dynamics. Time courses, two-way ANOVA with Tukey post hoc; single-time-point: one-way ANOVA with Tukey; VSA, median ± IQR; data, mean ± SEM. *p < 0.05; **p < 0.01; ***p < 0.001. SCI, spinal cord injury; Dex, dexmedetomidine; Inh, ERK inhibitor; ERK, extracellular signal–regulated kinase; BMS, Basso Mouse Scale; DPI, days postinjury; CMG, cystometrogram; EUS-EMG, external urethral sphincter electromyography.

Mitogen-activated protein kinase (MAPK) signaling and regulated necrosis are key contributors to secondary injury after SCI (Hu et al., 2023). To investigate their roles in Dex-mediated recovery, we examined how Dex and ERK Inh (LY3214996) modulate these pathways. Prior works showed that pERK1/2 activation rises in acute hours (Jin et al., 2020), whereas necroptosis signaling through RIP kinases can persist in the subacute window (Kanno et al., 2015). Accordingly, we surveyed pERK1/2 and RIPK1by Western blot at 3 h, 24 h, 3 d, 7 d, and 14 d after SCI and observed the distinct temporal dynamics across acute and subacute time points. Dex activated early ERK1/2 signaling and reduced subacute RIPK1 after SCI. Western blots showed that pERK1/2 was elevated at 3 h postinjury and further increased by Dex (245.7 ± 12.4% vs 184.7 ± 13.3%; *p* = 0.004), while ERK Inh blunted this effect (207.5 ± 11.7%; *p* < 0.01 vs Dex; Fig. 8*a*,*b*). By 7 d, RIPK1—indicative of necroptosis—was lowest in the Dex group (147.9 ± 10.9%), near Sham, and increased with ERK Inh (Dex vs Dex + Inh *p* = 0.005; Fig. 8*c*,*d*). To test whether ERK signaling contributes to Dex benefits, we compared Sham, SCI, SCI + Dex, SCI + Inh, and SCI + Dex + Inh in male mice. As with the other cohorts, hindlimb locomotion diverged over time (Fig. 9*a*). Dex rose steadily through 28 dpi (peak 5.00 ± 0.45) and remained elevated at 42 dpi (4.65 ± 0.50). Adding the inhibitor blunted this trajectory. SCI + Dex + Inh peaked lower at 28 dpi (3.32 ± 0.30; *p* = 0.02 vs Dex) and then declined, finishing at last time point markedly below Dex (2.63 ± 0.27; *p* < 0.01 vs Dex) and not different from SCI or SCI + Inh (*p* = 0.58 and *p* = 1.00; Fig. 9*b*). In other words, ERK Inh substantially reversed Dex's locomotor advantage, with the divergence most evident from 28 dpi onward (Fig. 9*a*,*b*). In the VSA evaluation, SCI showed fragmentation (many small, central spots show low emptying). Dex shifted toward physiology, while Dex + Inh countered the shift. Spot count fell with Dex to 19.0 [5.0–33.5] from 139.5 [32.0–243.0] in SCI but rebounded with SCI + Dex + Inh to 126.0 [45.8–277.8]. Voiding in corner increased with Dex to 40.41% [18.7–61.8] but was lower with SCI + Dex + Inh (26.76% [12.21–36.97]) and SCI + Inh (18.99% [16.02–22.62]). Large voids rose with Dex to 28.57% [14.2–65.5] from 1.85% [0.0–4.2] in SCI but were rare with SCI + Dex + Inh (0.00% [0.00–2.4]). VE improved with Dex to 80.31% [59.1–99.3], approximating Sham 83.88% [68.8–90.1] but fell toward injury with SCI + Dex + Inh (46.38% [9.5–62.6]) and means with SEM show in Supplementary Fig. 3*c*. PCA on VSA features explained 80.38% of variance (PC1 = 63.33%; PC2 = 17.04%). PC1 captured the pathological-to-physiological gradient (larger, corner voids and higher VE vs many small, central spots), with SCI at the pathological end and Sham at the physiological end, Dex shifted toward Sham, and both inhibitor arms shifted back toward SCI (Fig. 9*c*,*d*; Supplementary Fig. 3*c*). We also assessed CMG and EUS-EMG in this cohort at 42 dpi. The results showed that Dex + Inh reduced VE to 38.21 ± 13.48% (*p* = 0.01 vs Dex; *p* = 1.0 vs SCI). SCI + Inh 43.33 ± 8.7% also matched SCI (41.72 ± 7.8%). EMG showed that the sphincter SP during voiding in the Dex + Inh group was shorter (0.08 ± 0.02) and close to the SCI than to the Dex (0.12 ± 0.02; Fig. 9*e*). Latency to first void was longer with Dex + Inh and returned toward injury (357.80 ± 64.96 s vs SCI; *p* = 0.59). In both inhibitor arms, pathological NVCs were elevated (SCI + Dex + Inh 1.76 ± 0.36; SCI + Inh 1.71 ± 0.27), not different from SCI, and worse than Dex (*p* < 0.01). VA trended toward Sham with Dex (21.92 ± 3.05 cmH_2_O), and SCI + Dex + Inh (23.79 ± 1.62) remained intermediate (Table 3). PCA on the top five urodynamic contributors (VE, SP, first voiding latency, NVC, VA) explained 79.75% of the variance (PC1 = 66.39%; PC2 = 13.36%). PC1 reflected a continuum from coordinated, efficient emptying (longer SPs, higher efficiency, shorter latency, fewer NVCs) to delayed, fragmented patterns; PC2 primarily separated groups by pressure magnitude. Group positions followed biology. Sham and SCI anchored opposite ends of PC1, Dex shifted toward Sham, SCI + Dex + Inh moved back toward the injury side, and SCI + Inh remained near SCI. This multivariate structure mirrors the single-parameter findings that Dex normalizes coordination and emptying, while ERK Inh diminishes those gains (Fig. 9*f*).

**Table 3.** CMG and EUS-EMG parameters in male mice comparing Dex with ERK inhibitor

	Sham (*n* = 9)	SCI (*n* = 10)	SCI + Dex (*n* = 7)	SCI + Inh (*n* = 9)	SCI + Dex + Inh (*n* = 8)
CMG parameters					
VA, cmH2O	14.13 ± 1.36	25.79 ± 2.14**	21.91 ± 3.05	26.62 ± 3.54**	23.79 ± 1.62
CD, S	14.69 ± 0.82	22.15 ± 2.63	19.30 ± 1.91	20.37 ± 1.29	24.68 ± 3.17*
VI, S	86.88 ± 13.24	117.13 ± 22.26	143.08 ± 31.13	104.91 ± 52.67	71.93 ± 30.96
Voiding contraction, number/minute	0.91 ± 0.19	0.31 ± 0.06**	0.62 ± 0.19	0.24 ± 0.07**	0.20 ± 0.08**
NVC, number/minute	0.26 ± 0.09	1.47 ± 0.28**	0.34 ± 0.09^#,$$,^^^	1.71 ± 0.27**	1.76 ± 0.36**
Area IVP, cmH2O.S	97.40 ± 7.47	266.65 ± 35.42***	181.3 ± 18.83	233.49 ± 28.48*	251.67 ± 35.66**
Area IVP/bursting period, cmH2O	8.38 ± 0.99	34.23 ± 5.53*	5.68 ± 1.41^#^	24.8 ± 23.22	27.56 ± 9.82
First voiding, S	85.41 ± 14.40	466.19 ± 60.42***	204.45 ± 46.01^##, $$^	465.10 ± 41.59***	357.80 ± 64.96**
VE, %	87.22 ± 2.71	41.72 ± 7.77**	77.94 ± 4.81^#, $, ^^	43.33 ± 8.71**	38.21 ± 13.48**
EMG parameters during voiding bladder contraction					
Bursting period (S)	3.27 ± 0.53	0.92 ± 0.1**	2.98 ± 0.26^#^	2.33 ± 0.43	2.79 ± 0.61^#^
SP (S)	0.17 ± 0.01	0.06 ± 0.00***	0.12 ± 0.02*^, #, $^	0.06 ± 0.00***	0.08 ± 0.02***

Values are mean ± SEM. Group comparisons used one-way ANOVA with Tukey's post hoc; if variance was unequal, Welch's ANOVA with Games–Howell. Symbols mark post hoc differences: * versus Sham, ^#^ versus SCI, ^$^ versus SCI + Inh, ^ versus SCI + Dex + Inh; one, two, and three symbols denote *p* < 0.05, *p* < 0.01, and *p* < 0.001, respectively.

CMG, cystometrogram; EUS-EMG, external urethral sphincter electromyography; IVP, intravesical pressure; SCI, spinal cord injury; Dex, dexmedetomidine; Inh, inhibitor.

Collectively across BMS, VSA, and urodynamics, ERK pathway Inh reverted Dex's effects, consistent with ERK dependence of Dex-mediated locomotor recovery and restoration of bladder emptying and coordination.

## Discussion

### Pharmacological induction of early hypothermia

This strategy helps overcome key barriers that limit clinical use of systemic cooling after acute SCI. Although hypothermia is neuroprotective in preclinical models, its efficacy declines sharply beyond ∼3 h postinjury (Kabon et al., 2003; Che et al., 2011), whereas real-world initiation in humans is often delayed 6–8 h because of transport, stabilization, imaging, and surgical preparation (Levi et al., 2009; Cappuccino et al., 2010; Dididze et al., 2013). Surface cooling is slow and frequently insufficient, and although endovascular cooling cools more efficiently, it requires hospital placement, takes ∼3 h to reach target temperature, and carries procedural risks (Simosa et al., 2007). Cold-defense responses such as shivering further slow cooling and raise metabolic demand (Nakamura and Morrison, 2011), motivating interest in the pharmacological suppression of hypothalamic shivering pathways (Liu et al., 2015).

Dex with a strong safety profile during therapeutic hypothermia (Battaglini et al., 2022; Cocchi et al., 2025) shows stable pharmacokinetics (Mantecon-Fernandez et al., 2023) and minimal hemodynamic or respiratory depression (Lee, 2019). Dex lowers the shivering threshold via central α_2_-adrenergic mechanisms (Sallinen et al., 1997; Lahdesmaki et al., 2003; Callaway et al., 2015), inhibits excitatory descending pathways (Chiu et al., 1995), and reduces sympathetic tone (Talke et al., 1997), all facilitating cooling. We show that a single Dex dose given 1 h after SCI rapidly induces and sustains moderate hypothermia for ∼16 h, consistent with prior work (Pan et al., 2016). This pharmacological–physical strategy overcomes shivering-related limitations of conventional cooling and may enable earlier application within the critical therapeutic window.

Hypothermia duration is another determinant of efficacy, yet no consensus exists on optimal length. Preclinical SCI studies vary widely, with most in the 4 h range (Chatzipanteli et al., 2000; Dzsinich et al., 2000; Shibuya et al., 2004; Strauch et al., 2004; Tsutsumi et al., 2004; Duz et al., 2009; Horiuchi et al., 2009; Lo et al., 2009; Batchelor et al., 2011; Kao et al., 2011; Maybhate et al., 2012; Ok et al., 2012; Grulova et al., 2013; Saito et al., 2013; Bazley et al., 2014; Wang and Zhang, 2015; Sarkar et al., 2022), whereas human SCI protocols generally maintain hypothermia for ≥24 h (Cappuccino et al., 2010; Levi et al., 2010; Tripathy and Whitehead, 2011; Madhavan et al., 2012; Dididze et al., 2013). Prolongation beyond 48 h increases cardiovascular complications (Ahmad et al., 2014). In healthy humans, Dex's sedative effects dissipate within 2 h (Yatabe et al., 2016), but in SCI, trauma-related alterations in drug metabolism may extend its actions (Segal and Brunnemann, 1989; Garcia-Lopez et al., 2007), potentially explaining the sustained hypothermia we observed. This window overlaps with the acute secondary injury phase, maximizing neuroprotective potential.

Rewarming must be controlled, as rapid rewarming can trigger hyperkalemia, hypoglycemia, inflammatory activation, and loss of hypothermic benefit (Jacobs et al., 2013). Thus, controlled rewarming is essential for any Dex-based hypothermia protocol. In our study, mice gradually recovered from ∼16 h of Dex sedation and rewarmed passively at a safe rate of 0.23°C/h, closely matching optimal clinical rewarming recommendations (Vedantam and Levi, 2021).

### Potent therapeutic efficacy in the locomotor and urination functional improvement

CH produces only modest and short-lived locomotor improvements after SCI, ∼24% on average (Batchelor et al., 2013) and rarely yields benefits beyond 6 weeks (Lo et al., 2009; Wang and Pearse, 2015), particularly in severe injury models where effects are minimal (Westergren et al., 2000; Yu et al., 2000; Inamasu et al., 2003). Our head-to-head comparison confirmed this pattern: CH did not improve locomotion in a severe contusion model, whereas Dex-induced hypothermia significantly enhanced ambulatory recovery. The effect size was functionally meaningful, as Dex-treated mice achieved higher BMS scores than CH, representing categorical gains such as transitioning from partial to consistent weight-supported stepping with improved coordination.

Restoring bladder function is a major SCI priority, yet clinical hypothermia studies rarely report urinary outcomes because of motor-focused endpoints, limited urological measures, short follow-up, and the poor alignment of autonomic dysfunction with AIS grades (Alexander et al., 2009; Krassioukov et al., 2012; Wecht et al., 2021). Mechanistic links between hypothermia and micturition have also been underexplored. Here, we provide the first comprehensive assessment of hypothermia's effects on bladder recovery using urodynamics and urine-spot analysis. Dex increased VE by ∼30% and improved physiological markers, including longer EUS SPs, fewer NVCs, and shorter first-void latency, supported by greater raphe c-Fos activation and lumbosacral 5-HT preservation. Behavioral measures mirrored these effects, with fewer, larger, corner-localized voids. Together, these findings show that Dex-induced hypothermia more effectively restores integrated micturition circuits than surface cooling, yielding superior bladder and locomotor recovery.

Variability among Dex-treated mice is expected in contusive SCI, where differences in the spared tissue and descending pathways strongly shape recovery. Our correlations confirmed this, showing that serotonergic fiber density and raphe integrity predict voiding outcomes. Because bladder function is multidimensional, animals may improve in some parameters more than others, reinforcing the value of multivariate analyses such as PCA. Additional variability likely reflects individual pharmacological responsiveness and physiological state. Importantly, consistent patterns across animal batches and retention of all animals in analyses demonstrate that the treatment effect is robust despite inherent biological heterogeneity.

### Synergistic neuroprotection mechanism

**Figure 10.**
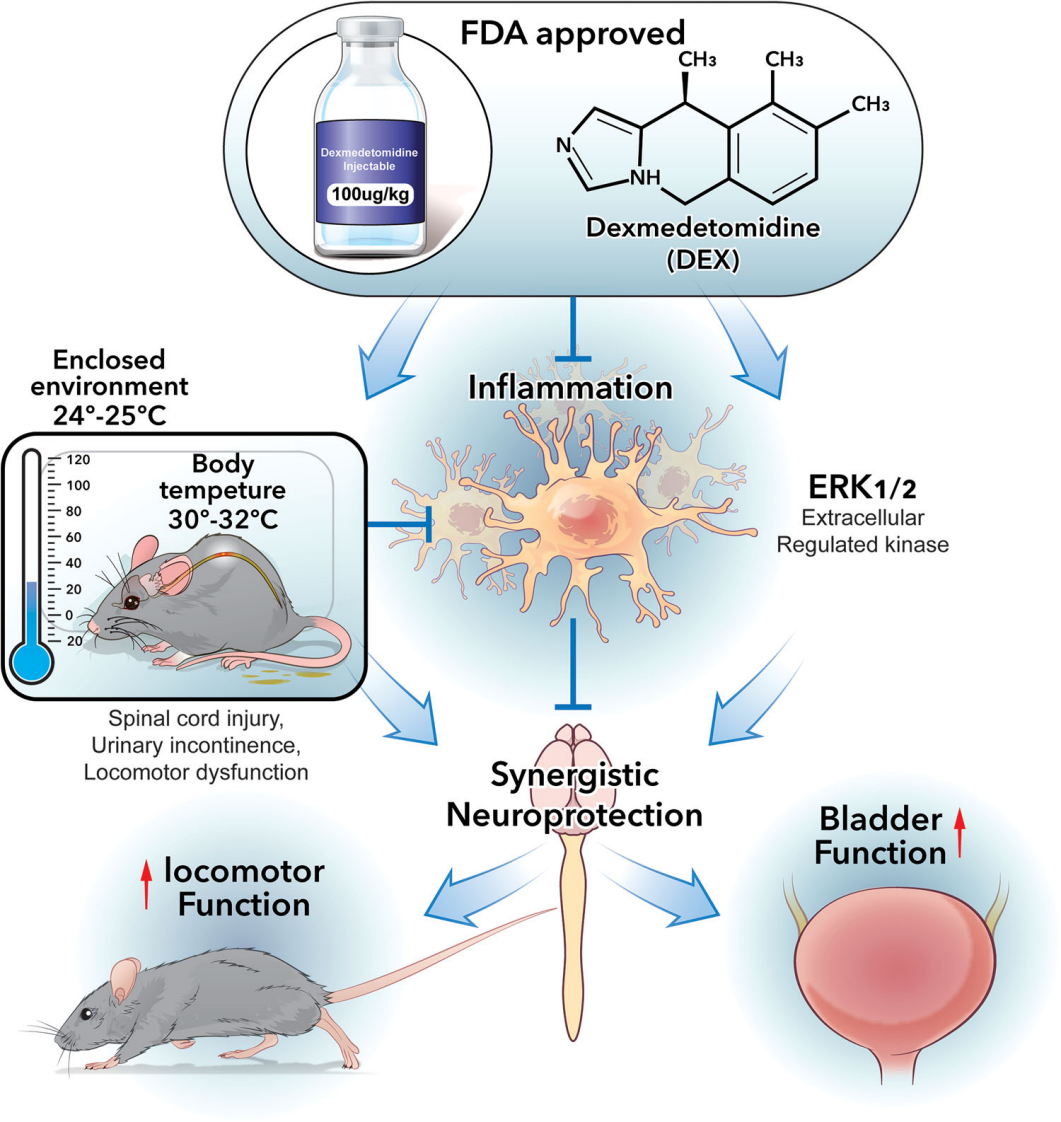
The synergistic neuroprotective mechanism of Dex-induced hypothermia in treating SCI. Schematic overview summarizing the synergistic neuroprotective mechanism of Dex-induced hypothermia based on the principal findings of this study. Dex-induced hypothermia and Dex intrinsic anti-inflammatory and ERK-dependent neuroprotective pathways convergently enhanced tissue sparing and functional restoration after SCI.

CH provides limited and short-lasting neuroprotection (Lee et al., 2007; Herlambang et al., 2011; Wang and Zhang, 2015; Wang and Pearse, 2015), whereas Dex also reduces cell death (Pan et al., 2016; Rodriguez-Gonzalez et al., 2016; Yin et al., 2018; Gao et al., 2019; Sun et al., 2019; Sun et al., 2020; Feng et al., 2021; Hu et al., 2022; Karakaya et al., 2022; Liu et al., 2022), offering protective actions that overlap with but extend beyond cooling (Fig. 10). This synergy likely involves ERK signaling (Shi et al., 2018; Teng et al., 2019), as ERK Inh reduced the benefits. Dex's anti-inflammatory effects further contribute, as an α_2_-adrenergic agonist [97–99], while hypothermia dampens early proinflammatory cytokines. Notably, Dex produced a delayed IL-10 rise at 3–7 dpi, accompanied by reduced macrophage density and blunted by rewarming, indicating a sustained proresolution environment that limits secondary damage. This coordinated anti-inflammatory response likely underlies the superior preservation of the spinal cord tissue supporting locomotor and bladder control.

### Safety of pharmacologically induced hypothermia

Although mild hypothermia is generally safe in SCI, both moderate cooling and Dex can cause bradycardia (Talke and Anderson, 2018), and hypothermia may impair respiration (Polderman, 2009; Nielsen et al., 2013; Ransom et al., 2022), warranting monitoring even though Dex rarely produces respiratory depression at clinical doses (Belleville et al., 1992; Gertler et al., 2001; Oguz et al., 2025). Prior reports support the safety of combining Dex with hypothermia (Cosnahan et al., 2021; Battaglini et al., 2022; Elliott et al., 2022; Naveed et al., 2022; Acun et al., 2024; Cocchi et al., 2025), but this had not been evaluated in SCI, so we continuously monitored vital signs until rewarming. As expected, bradycardia occurred but remained stable, sinus rhythm and SpO_2_ were maintained, and heart and respiratory rates normalized after rewarming. Infection risk was not increased, and Dex-treated mice exhibited fewer chronic complications and lower mortality than untreated SCI animals.

Despite its promise, several translational steps remain. Dex-induced hypothermia was initiated 1 h after injury for proof-of-concept, but clinically relevant windows are longer; delayed treatment (6–8 h) must be tested. The 100 µg/kg dose caused deep, prolonged hypothermia, which may pose cardiovascular risks in larger species, so dose optimization and titratable delivery are needed. Further validation in large-animal SCI models is required to assess pharmacokinetics, thermoregulation, and hemodynamic tolerance. Testing Dex in high-grade cervical SCI will also be essential. These steps are critical for advancing Dex-induced hypothermia toward practical clinical use.

In conclusion, Dex-induced early hypothermia provides strong neuroprotection after SCI, surpassing conventional cooling. By preserving supraspinal circuits, reducing lesion pathology, and improving both locomotor and bladder function through combined hypothermic, ERK-dependent, and anti-inflammatory mechanisms, Dex achieves broad functional benefit without cardiovascular or respiratory instability. These findings support Dex-induced hypothermia as a safe and clinically feasible acute intervention for SCI.
